# Tree Foliar Chemistry in an African Savanna and Its Relation to Life History Strategies and Environmental Filters

**DOI:** 10.1371/journal.pone.0124078

**Published:** 2015-05-20

**Authors:** Matthew S. Colgan, Roberta E. Martin, Claire A. Baldeck, Gregory P. Asner

**Affiliations:** Department of Global Ecology, Carnegie Institution for Science, Stanford, California, United States of America; University of Missouri, UNITED STATES

## Abstract

Understanding the relative importance of environment and life history strategies in determining leaf chemical traits remains a key objective of plant ecology. We assessed 20 foliar chemical properties among 12 African savanna woody plant species and their relation to environmental variables (hillslope position, precipitation, geology) and two functional traits (thorn type and seed dispersal mechanism). We found that combinations of six leaf chemical traits (lignin, hemi-cellulose, zinc, boron, magnesium, and manganese) predicted the species with 91% accuracy. Hillslope position, precipitation, and geology accounted for only 12% of the total variance in these six chemical traits. However, thorn type and seed dispersal mechanism accounted for 46% of variance in these chemical traits. The physically defended species had the highest concentrations of hemi-cellulose and boron. Species without physical defense had the highest lignin content if dispersed by vertebrates, but threefold lower lignin content if dispersed by wind. One of the most abundant woody species in southern Africa, *Colophospermum mopane*, was found to have the highest foliar concentrations of zinc, phosphorus, and δ^13^C, suggesting that zinc chelation may be used by this species to bind metallic toxins and increase uptake of soil phosphorus. Across all studied species, taxonomy and physical traits accounted for the majority of variability in leaf chemistry.

## Introduction

Leaf chemistry is fundamental to tree physiology [1–4], and has evolved in concert with other plant traits to serve plant functional strategies [5–10]. African savannas impose a unique set of selective pressures on the foliar chemistry of woody plants: drought, frequent fires and megaherbivores such as elephants and giraffe, and tree-grass interactions [11–13]. Chemical studies of savanna vegetation have largely focused on grasses to characterize forage quality for wild ungulates and herbivores [11,14–16] and for farmed livestock [17,18]. A foundational study of savanna woody plants found foliar condensed tannins, known to protect plant cells against microbial attack, to inactivate digestive enzymes in insect herbivores, and to slow digestion by mammalian herbivores [11]. There is debate in the literature as to whether a woody species dominant in southern Africa, *Colophospermum mopane*, generates secondary metabolites in response to herbivory [19–21]. Cost-benefit analyses have also indicated that leaf nitrogen (N) content is significantly lower and lignin content higher in leaves of savanna trees compared to other biomes, suggesting an investment in longevity and toughness at the expense of N-rich photosynthetic compounds, such as Rubisco and chlorophyll [22].

African savanna woody plants have classically been divided into two morphologically defined groups: fine-leaf and broad-leaf (Scholes and Walker, 1993). Fine-leaf savanna trees and shrubs, including the archetypal *Acacias* found throughout African savannas, exhibit spinescence and are typically capable of N-fixation. Broad-leaf savanna woody plants typically have fewer or no physical defenses but often utilize foliar chemical defenses, such as higher tannin concentrations [11]. Fine-leaf species are often found in clayey, poorly drained soils with higher nutrient concentrations in lowland areas, while broad-leaf species are more common on sandier, nutrient-poor soils found either on hill crests or alluvial deposits (Biggs 2003). Despite these general trends, the morphological trait of leaf size is not necessarily reflective of functional and chemical traits. Attributing differences in plant chemistry to environmental pressures and herbivore disturbance remains a challenge to the understanding of savanna woody plants [23,24].

Our understanding of the functional strategies of savanna trees can benefit from measuring a broad spectrum of both organic compounds and elemental mineral concentrations. Plants utilize a wide variety of compounds in their leaves to support multiple functional strategies, which can be grouped into three categories as described by Asner and Martin [25]: light capture and growth, structure and defense, maintenance and metabolism. Light capture-growth compounds include primary metabolites, such as photosynthetic pigments (chlorophyll, carotenoids, etc.) and immediate products of photosynthesis, such as soluble carbon compounds [26–29]. The stable isotope measurement of δ^13^C serves as a metric of water use efficiency, an important growth trait in semi-arid savannas. Structure-defense compounds include secondary metabolites, such as lignin and cellulose that support strength and longevity [30], as well as phenols and tannins for chemical defense against insects and other herbivores [31,32]. The third group includes mineral nutrients, such as Ca, K, Mg, Zn, Mn, B, Fe, required to support a wide variety of processes within the leaf, including cell wall construction, stomatal function, and protein synthesis. Although foliar chemistry varies regionally and globally with soil fertility and climate [29,33,34], how chemical investments of savanna tree species relate to their environment, herbivory, and genetics remains poorly understood.

Here we investigate 20 foliar chemical traits of 12 woody plant species in a semi-arid South African savanna, including the species in Kruger National Park (KNP) comprising 95% of woody biomass in the park. We assess the relative importance of environment (precipitation, temperature, geology, hillslope position) and two functional traits (physical defense and seed dispersal) to the chemical traits in addressing the following questions: i) Which leaf chemical traits best characterize these species? ii) How strongly do environmental factors relate to intra- and inter-specific variability in foliar chemistry? iii) Are the conflicting functions of discouraging leaf consumption while encouraging seed consumption reflected in the foliar chemistry of endozoochoratic species vs. species dispersed by other means, and if so to what degree and by which chemical compounds?

## Materials and Methods

### Study area

**Fig 1:**
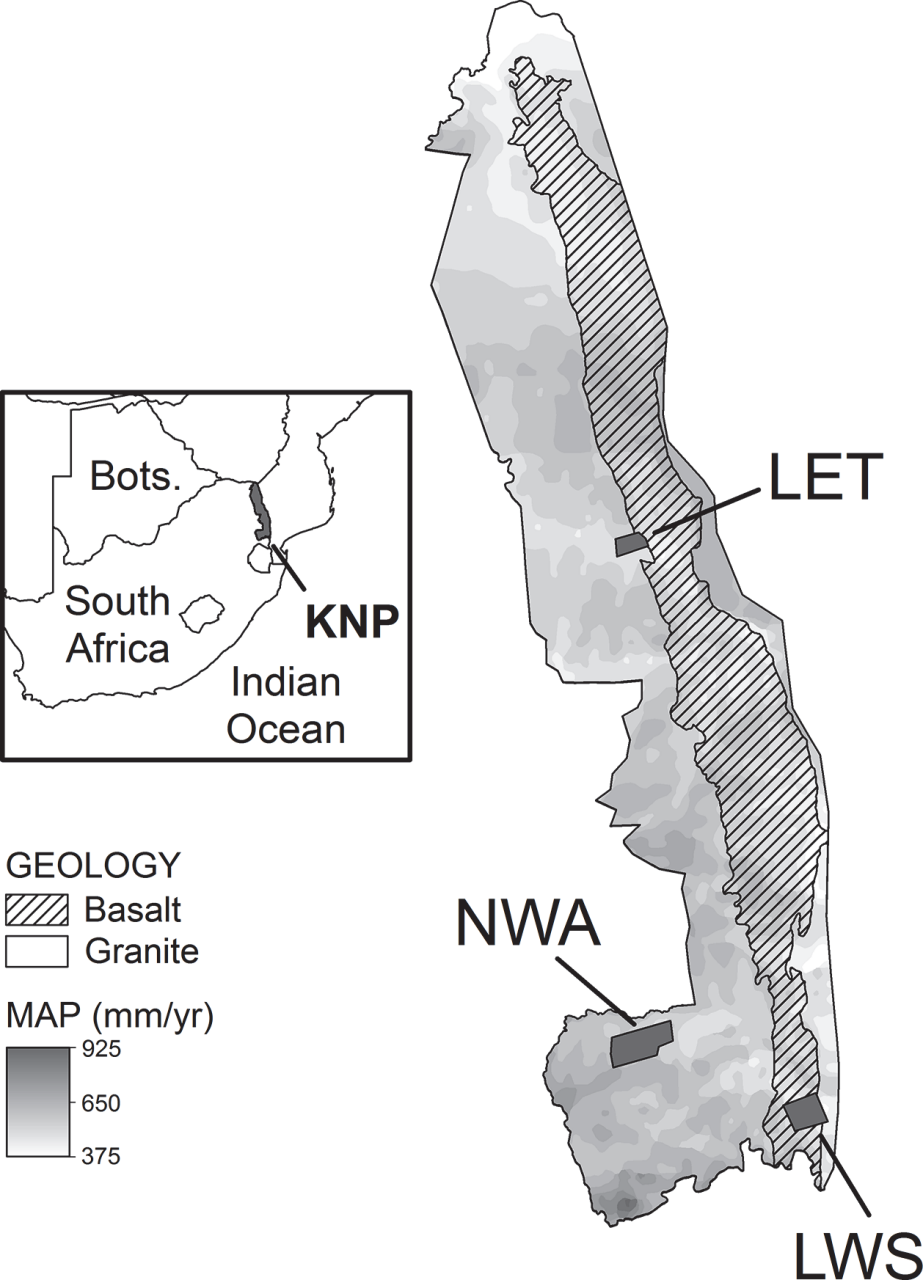
Site map. Sampling sites within Kruger National Park (KNP): Nwaswitshaka (NWA), Lower Sabie (LWS), and Letaba (LET). Inset shows location of KNP in South Africa.

Kruger National Park (KNP) is located in eastern South Africa, spanning an area 360 km north-south and 70 km east-west (Fig 1). KNP is roughly equally split into granite substrates in the west and basalt substrates in the east, with granites weathered to sandy, nutrient-poor soils and basalts weathered to clay-rich, primarily smectitic soils [35]. The climate of KNP is semi-arid with mean annual temperature and precipitation of 22°C and 550 mm∙yr^−1^, respectively, and an average potential evaporation of 7 mm∙day^−1^ [35]. KNP consists of semi-arid savanna vegetation with trees and shrubs interspersed with grass. Foliar sampling was conducted at three sites within KNP (Fig 1): Nwaswitshaka/Skukuza (NWA; S 25.02020°, E 31.48881°), Lower Sabie (LWS; S 25.21527°, E 31.94637°), and Letaba (LET; S 23.75635°, E 31.48559°). These sites were selected to cover geologic differences (granite and basalt substrate) and accompanying vegetation types, and within each site areas were selected to cover a range of hillslope topography (upland as defined as above seepline if visible or as on crest if not; lowland defined as below seepline).

### Foliar sampling

In March 2013, we collected top-of-canopy leaf samples from 238 individual trees at each of the three sites, across two hillslope locations per site (upland, lowland) (Table 1). Permission was granted by the South African National Park Service via the KNP Scientific Services office to collect leaf samples within KNP. Individual trees of common species were randomly sampled along 1–2 km transects within each site (Table 1), with 30–100 m separating each individual. The trees to be sampled were selected *a priori* using a crown species map generated from airborne imaging spectroscopy using methods described in Colgan et al. (2012), and species identity was verified in the field. This image-based species selection ensures measurement of foliar traits in species dominating the canopy community at the ecosystem scale, even though samples were collected in relatively small areas. Species dominance varied within and across sites. This accounts for the variation in the number of species measured at the different locations (Table 1). Sampled individuals were carefully selected to control for full sunlight canopies. Trees from all sites were distributed among five families, nine genera, and 12 species. The species sampled were *Acacia nigrescens* (Fabaceae), *Colophospermum mopane* (Fabaceae), *Combretum apiculatum* (Combretaceae), *Combretum imberbe* (Combretaceae), *Euclea divinorum* (Ebenaceae), *Sclerocarya birrea* (Anacardiaceae), *Terminalia sericea* (Combretaceae) with less abundant *Acacia tortilis* (Fabaceae), *Combretum hereroense* (Combretaceae), *Dichrostachys cinerea* (Fabaceae), *Diospyros mespiliformis* (Ebenaceae), *Spirostachys africana* (Euphorbiaceae). When possible, multiple individuals per species were collected at a given site and hillslope location to facilitate evaluation of intra- and inter-specific variation (Table 1).

**Table 1:** Site description by geology, precipitation, and woody plant species sampled (sample size in parantheses).

Site	Hillslope position	Geology	MAP (mm yr^-1^)	MAT (°C)	Species
NWA	Upland	Granite	553	22	*A*.*nig* (10), *C*.*api* (9), *S*.*bir* (10), *T*.*ser* (10)
NWA	Lowland & Riparian	Granite	553	22	*A*.*nig* (11), *A*.*tor* (5), *C*.*api* (10), *C*.*her* (3), *C*.*imb* (11), *D*.*cin* (10), *D*.*mes* (10), *E*.*div* (10), *S*.*afr* (10), *S*.*bir* (9), *T*.*ser* (2)
LET	Upland	Granite	458	23.3	*C*.*api* (10), *C*.*mop* (10)
LET	Lowland	Granite	458	23.3	*C*.*api* (13), *C*.*mop* (9), *E*.*div* (8)
LWS	Upland	Basalt	475	22	*E*.*div* (10), *S*.*afr* (10)
LWS	Lowland	Basalt	475	22	*A*.*nig* (10), *C*.*imb* (10), *D*.*cin* (10), *S*.*bir* (8)

Notes: MAP = Mean Annual Precipitation; MAT = Mean Annual Temperature; NWA = Nwaswitshaka (near Skukuza), LET = Letaba, LWS = Lower Sabie; *A*.*nig = Acacia nigrescens* (Fabaceae), *A*.*tor = A*.*tortilis* (Fabaceae), *C*.*api = Combretum apiculatum* (Combretaceae), *C*.*her = Combretum hereroense* (Combretaceae), *C*.*imb = Combretum imberbe* (Combretaceae), *C*.*mop = Colophospermum mopane* (Fabaceae), *D*.*cin = Dichrostachys cinerea* (Fabaceae), *D*.*mes = Diospyros mespiliformes* (Ebenaceae), *E*.*div = Euclea divinorum* (Ebenaceae), *S*.*afr = Spirostachys Africana* (Euphorbiaceae), *S*.*bir = Sclerocarya birrea* (Anacardiaceae), *T*.*ser = Terminalia sericea* (Combretaceae).

Leaf collections were conducted using pole-trimming techniques, and where not feasible, samples were obtained via slingshot. Only fully sunlit, mature leaves were taken and sealed in large polyethylene bags to maintain moisture, stored on ice in coolers, and transported to a local site for processing within 3 h, and usually less than 30 min. A photo was taken of each sample, which can be viewed at http://spectranomics.ciw.edu. A subset of leaves was selected from the branches for immediate weighing and extraction of leaf discs. Leaf disks were rapidly transferred to -80°C cryogenic containers and then to climate-controlled -80°C freezers until chemical assays were performed in the laboratory. The remaining leaves were detached from the branches and subsamples were selected to represent the range of colors and conditions found among all leaves collected. Occasionally, epiphylls were encountered, but these were removed prior to drying along with dust and other surface imperfections. These subsamples were dried at 70°C for a minimum of 72 h before vacuum sealing for transport to the laboratory for re-drying before chemical analysis. One voucher specimen was collected for each species, which are housed in the Carnegie Spectranomics Library (Department of Global Ecology, Carnegie Institution for Science, Stanford, CA USA).

### Chemical assays

Chemical analysis protocols, along with instrument and standards information, are downloadable from the Spectranomics website (http://spectranomics.ciw.edu). Dried leaves were ground in a 20 mesh Wiley mill and subsets were analyzed for a variety of elements and carbon fractions. Total element concentration of boron (B), calcium (Ca), potassium (K), magnesium (Mg), manganese (Mn), phosphorus (P), iron (Fe) and zinc (Zn) were determined by inductively-coupled plasma spectroscopy (ICP-OES; Therma Jarrel-Ash, Waltham MA) after microwave digestion in 10 ml concentrated (~70%) nitric acid solution (CEM MARSXpress; Matthews NC). Carbon (C) fractions including soluble C (composed of amino acids, pectins, simple sugars and starch), hemi-cellulose, cellulose and lignin were determined using sequential digestion of increasing acidity [36] in a fiber analyzer (Ankom Technology, Macedon NY). A subset of the ground material was further processed to a fine powder for determination of total C and N concentration by combustion-reduction elemental analysis (Costec Analytical Technologies Inc. Valencia, CA). A portion of the combustion gas from each sample was routed through an isotope ratio mass spectrometer (Finnigan S19, Thermo Scientific, Palm Beach FL) for determination of δ^13^C in the sample.

Frozen leaf disks, cryogenically preserved immediately following collection, were used to determine photosynthetic pigment (chlorophyll a, b and total carotenoid) as well as phenol and tannin concentrations. Pigment concentrations were measured using a UV-VIS spectrometer (Lambda 25, Perkin Elmer, Beaconsfield, UK) following digestion of frozen leaf tissue in 100% acetone [37]. phenol and tannin concentrations were determined colorimetrically using the Folin-Ciocalteau (Folin C) method following digestion and incubation in 95% methanol [38]. Polyvinylpyrrolidone (PVP) was used to bind the tannins in one portion of the sample such that the non-precipitable phenols could be measured and tannins quantified by subtraction [39]. These methods characterize the tannins that bind to PVP, and phenols sensitive to the Folin C reaction, rather than all known compounds as would be determined via high pressure liquid chromatography.

### Statistical analyses: NMDS, PCA, LDA

Nonmetric multidimensional scaling (NMDS) analysis is a non-parametric ordination technique used to quantify and visualize dissimilarity in a dataset, particularly for reducing high dimensional datasets into a lower dimensional space, typically two or three dimensions. NMDS is commonly used as an ordination method in community ecology [40] due to its robust nature and ability to capture non-linear dissimilarities among samples, in contrast to Principal Component Analysis (PCA), which is a linear transformation and retains the same number of dimensions as the original dataset.

Here we applied NMDS to investigate the chemical similarity among individual tree foliar chemistries, compressing the original dimensionality of 20 leaf chemical properties into two NMDS axes. Of the 238 samples collected, 219 samples were used for the NMDS analysis because two species (*A*.*tor* and *C*.*her*) were removed due to low sample sizes (*C*.*her* n = 3; *A*.*tor* n = 6). An additional ten samples were missing one or more chemical measurements and were removed. The NMDS analysis was executed using the “vegan” package of the software R [41]. The standard-normal standardization and Euclidean distance for dissimilarity [42] were used to transform the NMDS data prior to input because of differences in units and scale. A range of axes were tested (k = 2–10), all of which successfully converged although four axes had the lowest residual error (RMSE = 0.00028) with a stress metric of 0.092. The two-axis NMDS (RMSE = 0.00067, stress 0.21) was plotted to illustrate the dissimilarity even at just two axes.

Traditional linear analyses, PCA and linear discriminant analysis (LDA) were performed on the same samples included in the NMDS using JMP Pro (v 10.0.0, SAS Institute Inc., Cary, NC). Chemical traits (B, chlorophyll a+b, carotenoids, Ca, Fe, K, Mg, Mn, N and P) were log-transformed to improve normality.

### Classification and Regression Tree (CART) analysis

Chemical properties that were most representative of each species were identified using a type of Classification and Regression Tree (CART) [43] called a recursive partitioning (RPART) tree. RPART was used here to efficiently classify species using as few chemical properties as possible (i.e. to find a parsimonious classifier). The software packages “rpart” and “rpart.plot” of the software R (R-Core-Team 2012) were used to implement and display the univariate classification trees. All 238 collected samples were used in the CART analysis.

### Multivariate regression tree (MRT) analysis

A multivariate regression tree (MRT) is a similar statistical technique to CART, designed for multiple outputs. MRT was used here to understand how groups of chemical traits varied with environmental input variables (precipitation, hillslope position, and geology) and with functional traits (physical defense type and dispersal mechanism). MRT was selected over other methods for its ability to analyze complex chemical data that can include imbalance, nonlinear relationships, missing values, and high-order interactions. We used MRT to form clusters of chemical traits by repeatedly splitting the data, with each split defined by an environmental variable (e.g. precipitation < 550 mm yr^-1^) or a functional trait (e.g. long thorns). Each cluster represented an assblemage of chemical properties observed for a given environmental variable or functional trait (e.g. thorn type).

In a univariate regression tree, each split is chosen to minimize the sum of squares (SS) of the response variable about the node mean (and equivalently maximizes the between-node SS). The MRT is a multivariate extension of the regression tree. Again, each split wass chosen such that the SS within each resulting node was minimized; however, since the SS contained multiple response variables, computation of the SS necessitated that a mean of the multiple response variables be computed at each split. The measurement values were transformed as follows to permit computation of the multivariate mean at each node.

The chemical trait data were arranged with rows representing individual samples and columns containing each chemical trait. Each measurement was first divided by the column mean (i.e. mean of chemical trait, such as chlorophyll content), then the resulting values were each divided by the row mean (e.g. mean of relative concentrations for each sample). The row mean was necessary because the multivariate sum-of-squares can be skewed by a sample which had high relative concentrations for all chemical traits. This skew would be counter to the goal of grouping samples that have similar relative chemical compositions (e.g. similar ratios of lignin to cellulose). The procedure of normalizing by both column and row means had very minimal effect on the rank ordering of samples; see De’ath (2002) for further discussion. For comparison purposes, we tested our MRT analyses with and without normalization and found the recommended data normalization increased MRT performance, raising R^2^ values from 0.33 to 0.46.

The software package “mvpart” of the software R was used for all Multivariate Regression Tree (MRT) analyses (R-Core-Team 2012). Of the 238 samples collected, 227 were used for the MRT analyses because 11 samples had erroneous or missing hemicellulose or tannin values. Following De’ath (2002), the size of each tree was automatically determined by selecting the tree with the minimum value of the cross-validation error (see “plotcp” function in mvpart). The tree size was the same for all of the MRT analyses (five terminal nodes), resulting in a comparable level of complexity between analyses. The resulting trees also remained within one split of the minimum cross-validation error.

### Hillslope position and species variation in chemical traits

Species were measured on both hillslope positions (upland and lowland) at two sites, LET and NWA. At NWA, three species (*A*.*nig*, *C*.*api* and *S*.*bir*) were measured permitting an assessment of both the inter- and intra specific variation relative to hillslope position. Only a single species (*C*.*api*) was measured on both hillslope positions at LET. Interspecific and intraspecific variation in chemical traits were evaluated using standard least squares analysis of variance (ANOVA) at *p* < 0.05 level of significance in JMP. When needed, chemical traits were log transformed to improve normality.

## Results

### Interspecific variation in foliar chemistry

A summary of the 20 measured leaf chemical and elemental traits species is provided in Table 2, and the raw data for all 238 samples is contained in the CSV file “S1 Dataset”. Generally, we found relatively low inter-specific variation among primary metabolites. Specifically, chlorophyll a+b, carotenoid, and N concentrations had higher variances within species compared to between species. Species with consistently high primary metabolite concentrations were *A*.*nig* and *D*.*cin*, with related high N content and low C:N ratios.

**Table 2:** Mean (odd rows) and standard deviation (even rows) of 20 foliar chemical properties and two functional traits for 12 savanna woody plant species.

	*A*.*nig*	*A*.*tor*	*C*.*api*	*C*.*her*	*C*.*imb*	*C*.*mop*	*D*.*cin*	*D*.*mes*	*E*.*div*	*S*.*afr*	*S*.*bir*	*T*.*ser*
**Light & Growth**												
Chl a+b (mg g^-1^)	4.31	4.10	3.51	4.88	4.65	3.38	4.46	4.72	2.08	4.13	4.24	2.63
s.d.	1.60	1.64	0.87	0.82	1.34	0.47	1.43	1.48	0.72	1.03	0.97	0.38
P (%)	0.11	0.10	0.12	0.10	0.12	0.15	0.11	0.10	0.08	0.15	0.08	0.09
s.d.	0.02	0.01	0.03	0.01	0.02	0.04	0.03	0.01	0.01	0.04	0.02	0.01
N (%)	2.4	2.4	1.8	1.9	2.0	1.9	2.4	1.8	1.1	1.8	1.4	1.3
s.d.	0.4	0.4	0.3	0.2	0.4	0.2	0.5	0.3	0.2	0.2	0.3	0.1
δ^13^C	-29.2	-29.3	-27.9	-28.3	-28.2	-26.6	-28.2	-29.4	-28.9	-26.9	-29.0	-27.9
s.d.	0.9	1.5	0.9	1.0	0.6	0.8	0.8	0.5	1.0	0.6	1.0	0.3
Sol C (%)	50.7	70.8	66.2	63.5	64.5	55.3	49.2	56	51.1	84.8	59.1	48.3
s.d.	4.3	14.3	3.5	2.5	2.1	3.4	3.9	3.4	4.6	1.1	3.3	2.5
Water (%)	43.7	44.9	52.1	50.3	55.4	48.3	50.5	52.1	51.3	56.6	60.7	53.2
s.d.	15.1	4.9	3.6	0.6	3.6	2.4	4.5	2.9	3.4	1.4	2.3	2.7
Car (mg g^-1^)	1.01	0.98	0.80	1.09	1.11	0.86	1.18	1.14	0.51	0.89	0.96	0.67
s.d.	0.28	0.32	0.16	0.13	0.27	0.10	0.35	0.29	0.13	0.19	0.20	0.11
**Defense & Structure**												
Tannins (mg g^-1^)	35	74	52	43	35	38	49	46	59	61	65	65
s.d.	10	34	9	4	12	4	11	12	15	15	15	9
Phenols (mg g^-1^)	69	181	131	134	88	87	111	128	115	151	143	136
s.d.	21	81	21	6	19	10	29	26	21	29	27	16
Lignin (%)	20.3	13.7	10.5	17.6	10.6	23.8	31.1	25.8	33.1	4.1	28.8	30.4
s.d.	3.0	8.7	3.4	3.4	1.6	2.6	3.4	2.4	4.5	0.5	4.7	3.4
Cellulose (%)	15	6.8	14.8	12.1	13.4	12.1	11.3	12	14.3	5.5	10.8	16.9
s.d.	1.7	4.5	2.1	1.0	0.9	1.0	1.0	1.2	2.5	0.4	1.1	1.8
Hemicell (%)	14.0	8.5	8.5	6.9	11.5	8.7	8.4	6.2	2.0	5.7	1.7	4.0
s.d.	1.6	1.6	1.8	1.5	1.3	0.8	1.9	1.2	1.4	0.6	1.7	1.4
C (%)	46.9	51.0	49.3	50.2	46.5	49.8	50.2	50.5	50	44.4	47.2	48.7
s.d.	1.5	1.3	1.4	1.8	2.5	0.7	1.8	1.0	0.9	1.3	1.8	0.6
**Maintenance & Metabolism**												
Ca (%)	1.8	1.1	1.1	1.2	1.7	1.6	1.0	1.3	1.2	1.6	2.1	0.6
s.d.	0.7	0.4	0.4	0.7	0.3	0.3	0.3	0.4	0.4	0.4	0.7	0.1
K (%)	0.61	0.72	0.65	0.86	0.8	0.56	0.63	0.66	0.58	0.59	0.73	0.52
s.d.	0.15	0.12	0.15	0.28	0.14	0.10	0.18	0.13	0.18	0.21	0.18	0.1
Mg (%)	0.33	0.14	0.31	0.27	0.22	0.23	0.25	0.2	0.4	0.37	0.24	0.2
s.d.	0.09	0.04	0.08	0.05	0.05	0.06	0.06	0.06	0.08	0.08	0.06	0.05
Zn (μg g^-1^)	8.6	11.3	11.4	7.3	11.6	31.5	13.8	9.8	6.5	12.9	5.2	12.5
s.d.	1.4	1.7	3.0	2.6	3.3	6.9	2.7	1.6	1.5	2.9	1.6	1.9
Mn (μg g^-1^)	31	21	160	28	31	48	27	25	99	85	34	109
s.d.	10	2	154	12	17	20	10	6	70	68	82	27
B (μg g^-1^)	140	29	40	38	56	83	57	11	17	79	40	28
s.d.	36	11	10	13	26	16	17	4	3	16	13	6
Fe (μg g^-1^)	88	100	47	98	86	41	153	82	56	75	91	45
s.d.	29	27	12	16	48	10	111	18	26	24	32	12
**Non-foliar tree-level traits**												
Thorns	Short	Long	None	None	False	None	False	None	None	None	None	None
DispMech	En	En	A	A	A	A	En	En	En	B	En	A

Notes: Chl = Chlorophyll, Sol C = Soluble Carbon, Car = Carotenoids, Hemicell = Hemi-cellulose. A = Anemochory (wind), En = Endozoochory (ingestion by vertebrate animals), B = Ballochory (mechnically ejected). s.d. standard deviation, in units of each chemical. For species abbreviations, see Foliar Sampling section in Methods.

The largest inter-specific differences were observed in secondary metabolites, particularly lignin, cellulose, and hemi-cellulose concentrations. The *Combretum* species and *S*.*afr* had lower lignin than all other species. *E*.*div*, *D*.*cin*, and *T*.*ser* had the most lignin. *A*.*nig* had the lowest allocation to tannins and the highest hemi-cellulose. For compounds involved in maintenance and metabolism, Zn and B stood out sharply as higher for *C*.*mop* and *A*.*nig*, respectively.

### Chemical dissimilarity analysis and key identifiers of species

**Fig 2:**
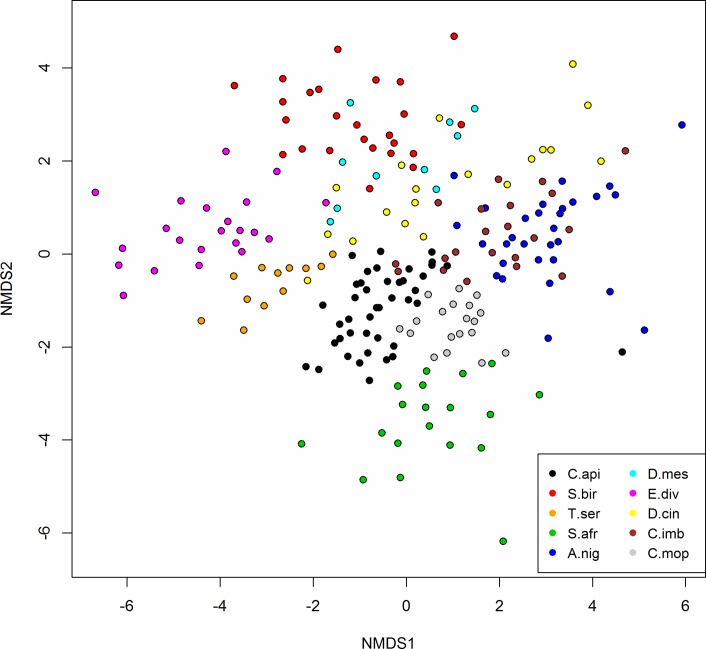
Nonmetric Multidimensional Scaling (NMDS) scatter plot showing dissimilarity in foliar chemistry between species. Points represent individual trees (n = 219), with 20 foliar chemical properties measured per tree. These properties were transformed using NMDS to two axes to illustrate the dissimilarity in foliar chemistry between species.

**Fig 3:**
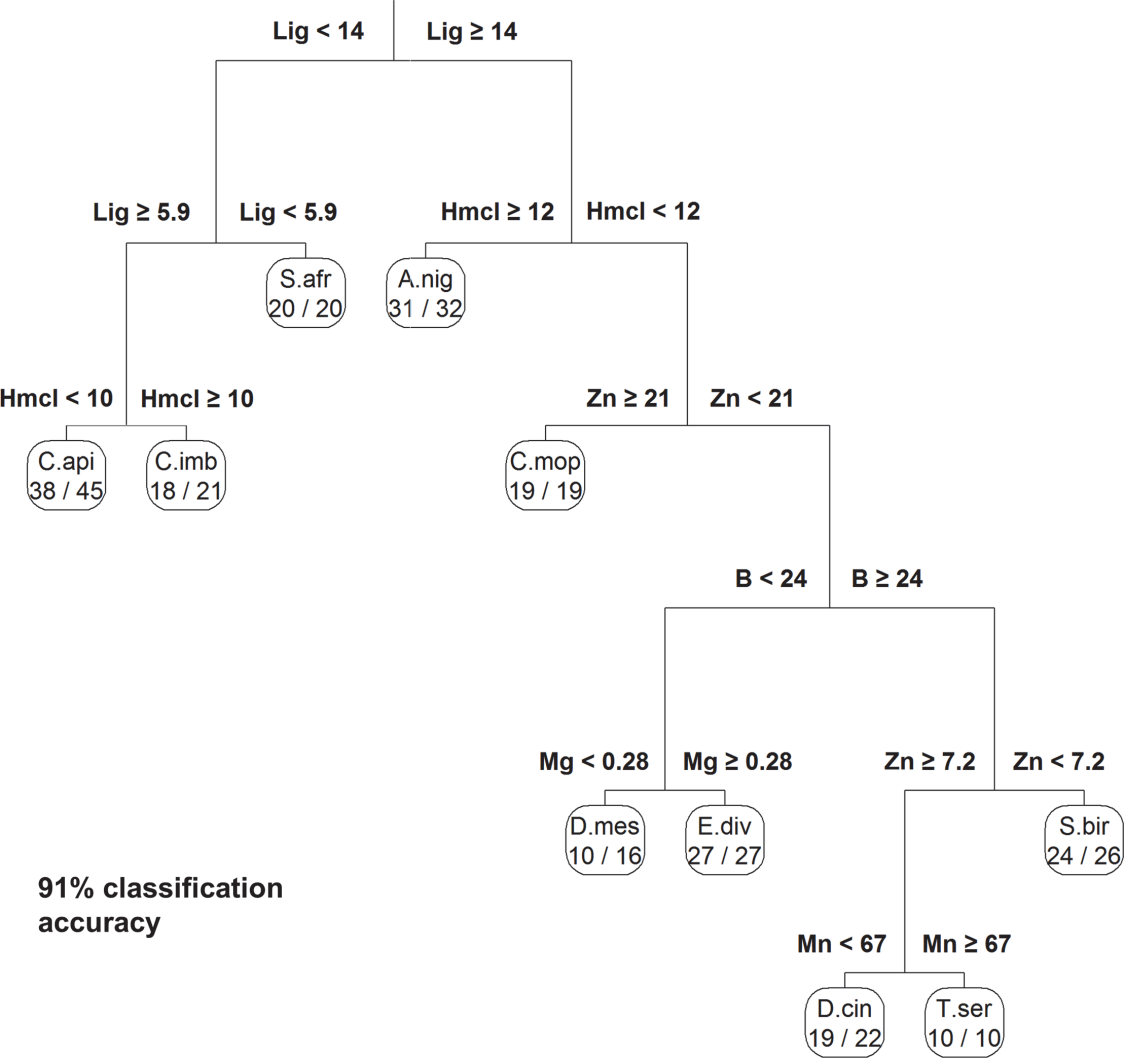
Classification and regression tree (CART) predicting species using chemical properties. All 20 foliar chemical and elemental properties measured for each tree sample (n = 238) were used as input to the CART algorithm. The algorithm selected six properties while retaining 91% classification accuracy. This analysis illustrates the minimum number of foliar chemicals needed to classify species and the relative importance of each trait in minimizing error in the classification (in descending order, from top to bottom). The equation above each branch indicates the chemical concentration used to perform the split (e.g. “Lig < 14” means samples with lignin concentrations less than 14% by mass). The units of concentration varied for each trait as follows (all were on a mass basis): Lig = lignin (%), Hmcl = hemi-cellulose (%), Zn = zinc (μg g^-1^), B = boron (μg g^-1^), Mg = magnesium (%), Mn = manganese (μg g^-1^). Numbers below species indicate the number of correct classifications divided by the total number of samples for that species. See text for key to species abbreviations.

The degree to which species vary chemically was assessed using a NMDS analysis, which reduced the 20 chemical traits of each individual tree to two NMDS axes. Plotting these transformed chemistries for each individual tree (Fig 2) revealed clusters closely aligned with species, without prior knowledge of taxonomic identification. Five species were chemically distinct from one another and from all other species: *C*.*api*, *C*.*mop*, *T*.*ser*, *E*.*div*, *S*.*afr*. Another three species exhibited some overlap with each other in NMDS space, but were distinct from the rest of the group: *S*.*bir*, *D*.*cin*, and *D*.*mes*. Two species were chemically indistinguishable in NMDS space: *A*.*nig* and *C*.*imb*. We then determined which chemical traits varied most between species and which subset of traits was the best predictor of species. Six chemical traits classified species with 91% accuracy (Fig 3): lignin, hemi-cellulose, Zn, B, Mg, and Mn (listed in descending order of importance for maximizing accuracy). The traditional linear methods, PCA and linear discriminant analysis (LDA) are presented in (S1 and S2 Figs). Each PC value contained significant portion of the variation (*p* < 0.001; χ^2^), however break points in the slope of the variance curve can be seen at 5 and again at 8 PCs, where 72% and 85% of the cumulative variation is explained. About 95% of the variation in chemical traits was explained with the inclusion of 12 PCs. LDA accurately classified 98% of the species using six chemical traits similar to those selected in the CART analysis: lignin (53%), soluble carbon (72%), boron (86%), zinc (88%), hemi-cellulose (95%), and manganese (98%).

### Multivariate correlations

**Fig 4:**
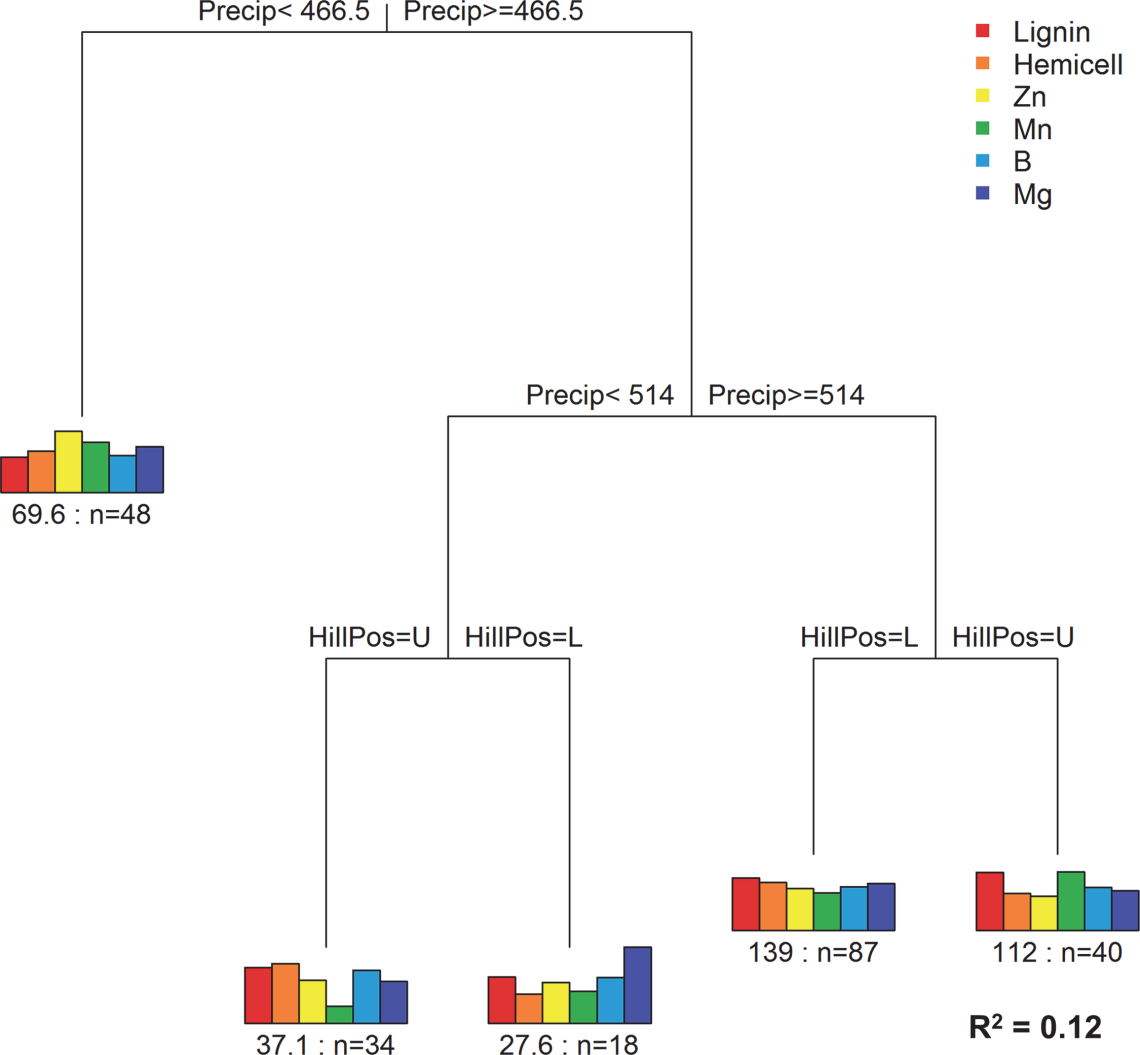
Multivariate regression tree (MRT) relating environmental variables to foliar chemical properties. The six foliar chemical response variables selected during the CART analysis (lignin, hemi-cellulose (Hemicell), Zn, Mn, B, Mg) were predicted using three environmental input variables (precipitation, hillslope position, and parent material). These environmental factors explained 12% of the total variance among these chemical properties. The column plots show the mean chemical concentrations of each cluster. n = number of individual tree samples; the value before n is the sum of squared errors (post-normalization) of chemical concentrations for that group.

Precipitation, parent material, and hillslope position accounted for 18% of total variance across all chemical traits and for 12% of the total variance across the six chemical traits identified to be most correlated to species (lignin, hemi-cellulose, Zn, B, Mg, and Mn) (Fig 4). The first split in the regression tree occurred at annual precipitation levels < 467 mm yr^-1^, which identified a group of individuals with a mean Zn concentration higher than the other four terminal-node groups. This group also had the lowest mean lignin concentration. The second split further delineated precipitation levels at 514 mm yr^-1^, and both of the third splits were based on hillslope position. The model did not select geology since the recursive algorithm achieved minimum relative error using only precipitation and hillslope position (i.e. to achieve the most parsimonious model).

**Fig 5:**
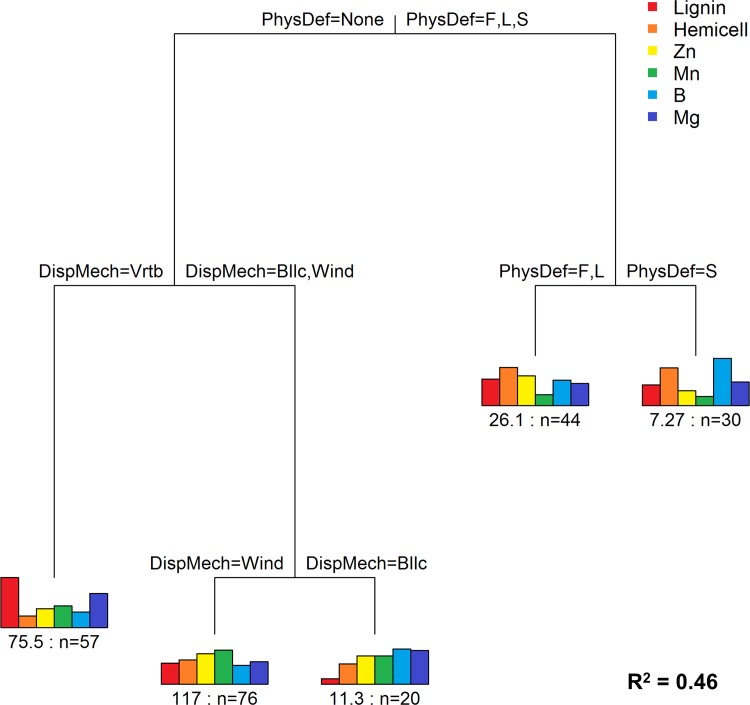
Multivariate regression tree (MRT) relating thorn type and seed dispersal to foliar chemical properties. Thorn type and seed dispersal mechanism accounted for 46% of total variance across six foliar chemical properties (lignin, hemi-cellulose (Hemicell), Zn, Mn, B, Mg). Note the increased variation between clusters here compared to Fig 4, indicative of better separation of dissimilar groups by defense and dispersal type. Physical defense types: F = false thorn, L = long thorn, S = short thorn, None. Dispersal mechanisms: Vrtb = dispersed via vertebrate ingestion (endozoochory); Bllc = mechanical ejection (ballochory); Wind = wind dispersed (anemochory). n = the number of individual plants; the value before n is the sum of squared errors (post-normalization) of chemical concentrations for that group.

Two functional traits associated with life history strategy, seed dispersal mechanism and physical defense, accounted for 46% of total variance (MRT analysis) across the six chemical response variables (Fig 5). The first split was driven by the presence/absence of thorns, followed by seed dispersal via endozoochory (ingestion by vertebrate animals). For comparison, a separate MRT analysis showed that species (as a qualitative predictor variable) explained 54% of the variance across the six chemical traits (S3 Fig). Although we expected that species ID would be the strongest predictor, since it was the criteria by which the six chemical traits were selected, we nonetheless performed this analysis to provide a benchmark for the dispersal and defense results.

### Hillslope position and species variation

At two sites, Letaba and Nwaswitshaka (NWA), replicate species were measured on both hillslope positions (upland and lowland). In general, species variation was independent of topographic position. At the Letaba site, all foliar chemical traits except for Chl a+b, K, and Fe displayed significant intra-specific variation and there was no interaction among species and hillslope position (Table 3). Seven foliar chemical traits were significantly different in upland and lowland positions at the Letaba site. Foliar traits related to light capture and growth (water, chl a+b, car and N content) as well as cellulose were higher on the lowland hillslope position. The micronutrients of Mn and Zn were lower in the lowland positions.

**Table 3:** Analysis of foliar chemical variation with hillslope position.

	Letaba (C.api)			Nwaswitshaka (A.nig, C.api, S.bir)		
	Species	Hillslope Position	Species × Position	Species	Hillslope Position	Species × Position
**Light & Growth**						
Chl a+b	-1.1	2.7*	1.0	3.0*	0.5	0.7
P	-2.7*	-1.6	-0.6	25.4*	0.4	2.5
N	-3.9*	2.5*	-0.4	64.5*	-0.8	0.3
d13C	-3.1*	0.2	-2.3*	6.5*	1.1	0.5
Sol C	12.2*	-2.0	-0.9	80.7*	-0.8	3.4*
Water	2.9*	3.7*	-0.4	18.9*	-0.1	0.1
Car	-3.8*	3.0*	1.5	5.8*	-0.5	1.1
**Defense & Structure**						
Tannins	6.4*	-1.6	1.1	49.8*	-1.9	2.9*
Phenols	7.3*	-0.9	1.4	54.2*	-1.8	1.2
Lignin	-22.8*	1.5	0.0	158.7*	-0.6	0.9
Cellulose	6.1*	2.8*	1.7	71.5*	4.3*	10.0*
Hemicellulose	-2.4*	-2.0	0.8	271.6*	-0.3	1.2
C	-4.1*	0.6	0.4	32.9*	-3.2*	0.2
**Maintenance & Metabolism**						
Ca	-4.1*	0.6	0.0	30.0*	1.0	1.6
K	1.7	0.4	-1.0	8.2*	-0.9	2.2
Mg	6.0*	1.8*	-1.0	8.0*	1.6	2.6
Zn	-14.4*	-2.6*	1.7	80.6*	-0.2	2.9*
Mn	5.5*	-4.3*	0.3	107.7*	-3.2*	9.1*
B	-11.2*	1.5	-0.9	123.6*	0.4	2.3
Fe	1.0	-0.6	2.6*	21.1*	-1.6	1.8

Results of analyses of variance (ANOVA) for foliar chemical and elemental properties at Letaba and Nwaswitshaka (table contains F-values). This analysis tests for differences within and among species, hillslope position, and their interactions. Not all species were present at both upslope and downslope locations; the species present at both and analyzed here are given in parentheses after the site name. Significant differences (*p* < 0.05) are indicated by the asterisk. Inter-specific differences at NWA are shown in S1 Table of SOM.

Foliar traits at the NWA site also displayed a high degree of inter-specific variation, but far fewer traits differed with hillslope position. Notably, cellulose and Mn followed the same pattern as measured in Letaba. Nearly all chemical traits displayed significant intra-specific variation for each of the three species at the NWA site (S1 Table). Very few traits showed significant interactions between inter-species and hillslope positions. Boxplots of inter-specific variation in foliar chemistry by hillslope position and site are additionally illustrated in S4–S7 Figs.

## Discussion

Our results reveal high levels of foliar chemical diversity among coexisting savanna woody plant species, suggestive of divergent life strategies. The largest differences in leaf chemical traits between species occurred for lignin, hemi-cellulose, Zn, and B (and to a lesser extent Mg and Mn). We found that environmental filters were weakly correlated with these chemicals (R^2^ = 12%), whereas two non-foliar traits—thorn type and seed dispersal mechanism—accounted for nearly as much variance in these chemicals as did species (R^2^ = 46% for thorn+dispersal, R^2^ = 54% for species). We found that three functional groups of woody plants in African savannas had a unique portfolio of lignin, hemi-cellulose, Zn, and B.

One such grouping is species with seeds dispersed by vertebrates (endozoochoratic), and which also have no physical defenses, *S*.*bir*, *D*.*mes*, *E*.*div*, had high lignin concentrations (26–33%) and lower hemi-cellulose (1.7–6.2%) in our study. These three species overlap in their primary dispersers, which in southern Africa are ungulates (e.g. antelope, elephants, giraffe), and for *D*.*mes* additionally birds and baboons [44,45]. Lignin and hemi-cellulose concentrations were not significantly correlated with the studied environmental gradients (substrate, rainfall, hillslope position, or site).

A second functional strategy in savanna trees is that of maximizing foliar growth via N-fixation, and then using primarily thorns to slow rates of mammalian herbivory, with minimal investment in tannins. Tannins are a subset of phenols, a broad group of non-structural defense compounds. Cooper and Owen-Smith [11] demonstrated that foliar condensed tannins can deter mammalian browsers while other types of tannin do not [46]. In the current study only hydrolysable tannins were measured. These are generally used as an indicator of defense against insect herbivory, so we draw on other studies investigating condensed tannins and mammalian herbivory in these tree species. Condensed tannin concentrations above 5% of dry matter have been shown to deter most mammalian herbivores, whereas hydrolysable tannins deter insect herbivore by inactivating digestive enzymes but typically do not deter mammalian browsers [11]. We found that the species with physical defenses (*A*.*nig*, *A*.*tor*, and *D*.*cin*) had the highest foliar N concentrations, highest N:P, and lowest C:N, among the woody species studied (all three species are mimosoid legumes). Cooper and Owen-Smith [11] found these three species are also strongly preferred by two mammalian browsers common in KNP, impala (*Aepyceros melampus*) and kudu (*Tragelaphus strepsiceros*), due to low condensed tannin concentrations (~0%, 3%, 3% of dry matter for *A*.*nig*, *A*.*tor*, and *D*.*cin*, respectively), as measured by Cooper and Owen-Smith [11]). To protect their nutrient investment, these species invest in physical defenses adapted to deter specific mammalian foliovores: hooked short thorns (<1 cm in length) of *A*.*nig*, which deter kudu more than impala; straight long thorns (up to 8 cm) of *D*.*cin*, which deter impala more than kudu; and both straight-long thorns and hooked-short thorns of *A*.*tor*, which deter kudu, impala, and giraffe [11].

Recent work by Cramer et al. [47] has shown that African savanna N-fixers, such as *A*.*nig* and *A*.*tor*, do not necessarily fix N to meet a high N demand, but rather to compete with C4 grasses during the tree seedling and sapling growth stages. The N content observed here was nearly identical between *A*.*nig*, *A*.*tor*, and *D*.*cin* (2.4 ± 0.4, 2.4 ± 0.4, 2.4 ± 0.5), in line with the hypothesis that N-fixation serves to maintain a nominal level of growth compounds. A primary difference observed here between the two N-fixers (*A*.*nig* and *A*.*tor*) was a fourfold higher B concentration in *A*.*nig*. Boron has long been known to be key to plant growth [48,49], particularly in cell wall development and metabolism, but also more recently B has been found to play a role in N-fixation [50], both in nodule cell wall structure [51] and as a signaling mechanism between bacteria and legumes [52–54]. We speculate that the higher levels of B observed in *A*.*nig* are indicative of active N-fixation, although further research is needed to investigate varying demands for boron among N-fixers.

A third “low cost” functional strategy comprises the majority of the trees in KNP, which are wind dispersed, have no physical defenses, and have varying amounts of chemical defenses. This strategy encompasses the members of the family Combretaceae studied here (*C*.*api*, *C*.*imb*, *C*.*her*, *T*.*ser*), as well as *C*.*mop* (Fabaceae), the most abundant tree in KNP and the one member of the Fabaceae studied here which does not form root nodules for N-fixation (although it has been argued the presence of cluster roots in *C*.*mop* serves a similar role; see Jordaan et al. [55]). We observed *C*.*mop* to have significantly lower N content (1.9 ± 0.2) than the other Fabaceae under study (*A*.*nig*, *A*.*tor*, *D*.*cin*), yet it has the highest foliar P and water-use efficiency (as indicated by δ^13^C measurements) than all other species studied. Surprisingly, we found high foliar Zn concentrations in *C*.*mop*, consistently three-fold higher than the next highest species (*D*.*cin* 13.8 ± 2.7 μg/g). Zinc is important in leaf development, and is also used in the chelating of toxic anions in soils for storage in leaf vacuoles [50]. We hypothesize that *C*.*mop* utilizes the process of Zn chelation to bind metallic toxins from the soil (e.g. Al and/or Fe oxides), possibly permitting increased uptake of soil P. In light of the findings of Cramer et al. [47] that tree seedling establishment may be P-limited when competing with grass, increased P uptake via Zn chelation may enable *C*.*mop* to better compete with grasses than does *A*.*nig* and other N-fixers. Further research is needed to confirm the competitive advantages that Zn chelation may afford *C*.*mop*, such as measurements of soil Zn content beneath *C*.*mop* canopies and accompanying measurements of photosynthetic rate of *C*.*mop* and other tree species during early growth stages.

An outlier species was *S*.*afr*, whose foliar defense is a latex exuded from leaves and under-bark which is toxic to many mammals [56], and whose dispersal mechanism is ballochory (the seed “explodes” from the fruit by dehiscence and squeezing). Consequently, the foliar chemistry of *S*.*afr* was dominated by a high soluble-C content (85 ± 1% of dry mass) and low intra-specific variation in most other foliar chemicals. Although *S*.*afr* is the only species studied from the family Euphorbiaceae, the use of a chemical deterrant to deter mammals is similar to that of the Combretaceae and other anemochorous taxa.

## Conclusions

We investigated the relationships between foliar chemical traits and i) environmental factors, ii) seed dispersal mechanisms, and iii) physical defense types in savanna woody plants. Our results reveal high levels of foliar chemical diversity among coexisting savanna woody plant species, suggestive of divergent life strategies. The greatest differences in leaf chemical traits between species occurred for lignin, hemi-cellulose, Zn, and B, and to a lesser extent Mn and Mg. We propose that portfolios of chemical traits such as these provide insight into functional strategies of savanna woody plants. One of the most abundant woody species in southern Africa, *Colophospermum mopane*, was found to have very high levels of Zn coupled with the highest observed P concentration and highest δ^13^C (proxy for water-use efficiency). Future work is needed to determine whether Zn chelation or another mechanism is responsible for the high Zn concentrations found in *C*.*mopane*, and whether this is linked to increased uptake of soil P and improved competition with grasses.

## Supporting Information

S1 Fig Principle component analysis (PCA). (A) Principle components (PC) plot showing the relationship of species to PC1 and PC2 derived from the ordination of 20 chemical traits. Points are colored by species. (B) Biplot showing the eigenvectors loadings of the 20 chemical traits on PC1 and PC2. (C) Scree plot showing the percent variation of the 20 chemical traits in the eigenvalue of each PC. Each value contained significant portion of the variation (*p* < 0.001; χ^2^), however break points in the slope of the curve can be seen at 5 and again at 8 PCs, where 72% and 85% of the cumulative variation is explained. 95% of the variation was explained with the inclusion of 12 PCs.
(TIF)

S2 Fig Linear discriminant analysis (LDA). Canonical plot showing the classification of species based on six chemical traits selected through stepwise linear discriminant analysis. Points are colored by species. The circles represent 95% confidence limits in prediction of the species mean value. Classification accuracy reached a plateau at 98% using six chemical traits. The traits entered the model in the following order (% classified follows in parentheses), lignin (53%), soluble carbon (72%), boron (86%), zinc (88%), hemi-cellulose (95%), and manganese (98%).
(TIF)

S3 Fig Multivariate regression tree using species as the sole predictor variable. Five groups of species accounted for 54% of the total variance across six chemical traits (lignin, hemi-cellulose (Hemicell), Zn, Mn, B, Mg). Although it was expected that species would have the strongest multivariate correlation to these six chemicals (because they were selected for this reason during the CART analysis in Fig 2), it is interesting to note that the combination of thorn type and dispersal mechanism have a similarly high correlation (46% variance explained; Fig 5). A total of seven predictor variables (hillslope position, precipitation, parent material, elevation, physical defense, dispersal mechanism, species) were the initial input to the recursive regression tree, but species was the strongest predictor (lowest SSE) for all splits. n = the number of individual plants; the value before n is the sum of squared errors (post-normalization) of chemical concentrations for that group.
(TIF)

S4 Fig Boxplots of 7 foliar chemical properties, including primary metabolites and other chemical properties related to light capture and growth. Chemical concentrations (by mass) are grouped by species, then site, and finally hillslope position. Car = carotenoids; Chl a+b = sum of chlorophyll a and chlorophyll b concentrations. X-axis labels follow the format: species_site_hillslopePosition,with NWA = Nwaswitshaka, LET = Letaba LWS = Lower Sabie; U = upland, L = lowland.
(TIF)

S5 Fig Boxplots of 6 foliar chemical properties, including secondary metabolites and other properties related to structure and defense. Chemical concentrations (by mass) are grouped by species, then site, and finally hillslope position. C% = soluble carbon. X-axis labels follow the format: species_site_hillslopePosition,with NWA = Nwaswitshaka, LET = Letaba LWS = Lower Sabie; U = upland, L = lowland.
(TIF)

S6 Fig Boxplots of 7 foliar elemental properties related to maintenance and metabolism. Elemental concentrations are grouped by species, then site, and finally hillslope position. X-axis labels follow the format: species_site_hillslopePosition, with NWA = Nwaswitshaka, LET = Letaba LWS = Lower Sabie; U = upland, L = lowland.
(TIF)

S7 Fig Boxplots of foliar carbon:nitrogen and nitrogen:phosphorus concentrations (by mass). C, N, and P concentrations were measured on a mass basis (see S4 and S5 Figs) in order to compute these fractions. The fractions are grouped by species, then site, and finally hillslope position. X-axis labels follow the format: species_site_hillslopePosition, with NWA = Nwaswitshaka, LET = Letaba LWS = Lower Sabie; U = upland, L = lowland.
(TIF)

S1 Table ANOVA results of chemical variance across species and hillslope position. Results of the intra-specific variation portion of analyses of variance (F-values) for leaf traits of canopy populations at Nwaswitshaka, showing differences for within species and hillslope position interactions. Significant differences (*p* < 0.05) are indicated by the asterisk.
(DOCX)

S1 Dataset Foliar chemical measurements. Dataset in CSV format containing the 20 chemical measurements (columns) for each of the 238 tree samples (rows), starting with “Chl-a (mg/g)” and ending with “Lignin (%)”. This file also contains species, hillslope position, date of collection, and other ancillary data.
(CSV)
