# Supplementary figures and images for: Tree Foliar Chemistry in an African Savanna and Its Relation to Life History Strategies and Environmental Filters
Source: PLoS One. 2015 May 20;10(5):e0124078. doi: 10.1371/journal.pone.0124078 (PMC4438986; doi:10.1371/journal.pone.0124078)

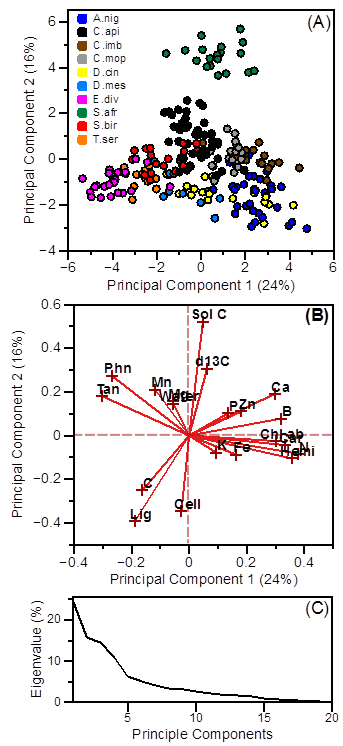

Supplement: S1 Fig — (A) Principle components (PC) plot showing the relationship of species to PC1 and PC2 derived from the ordination of 20 chemical traits. Points are colored by species. (B) Biplot showing the eigenvectors loadings of the 20 chemical traits on PC1 and PC2. (C) Scree plot showing the percent variation of the 20 chemical traits in the eigenvalue of each PC. Each value contained significant portion of the variation (p < 0.001; χ2), however break points in the slope of the curve can be seen at 5 and again at 8 PCs, where 72% and 85% of the cumulative variation is explained. 95% of the variation was explained with the inclusion of 12 PCs. (TIF) [file pone.0124078.s001.tif]

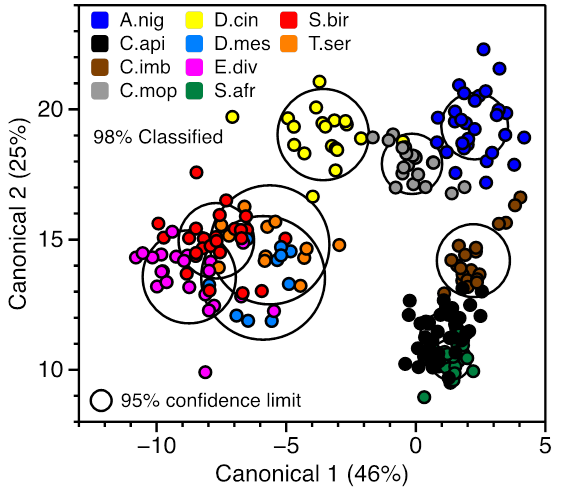

Supplement: S2 Fig — Canonical plot showing the classification of species based on six chemical traits selected through stepwise linear discriminant analysis. Points are colored by species. The circles represent 95% confidence limits in prediction of the species mean value. Classification accuracy reached a plateau at 98% using six chemical traits. The traits entered the model in the following order (% classified follows in parentheses), lignin (53%), soluble carbon (72%), boron (86%), zinc (88%), hemi-cellulose (95%), and manganese (98%). (TIF) [file pone.0124078.s002.tif]

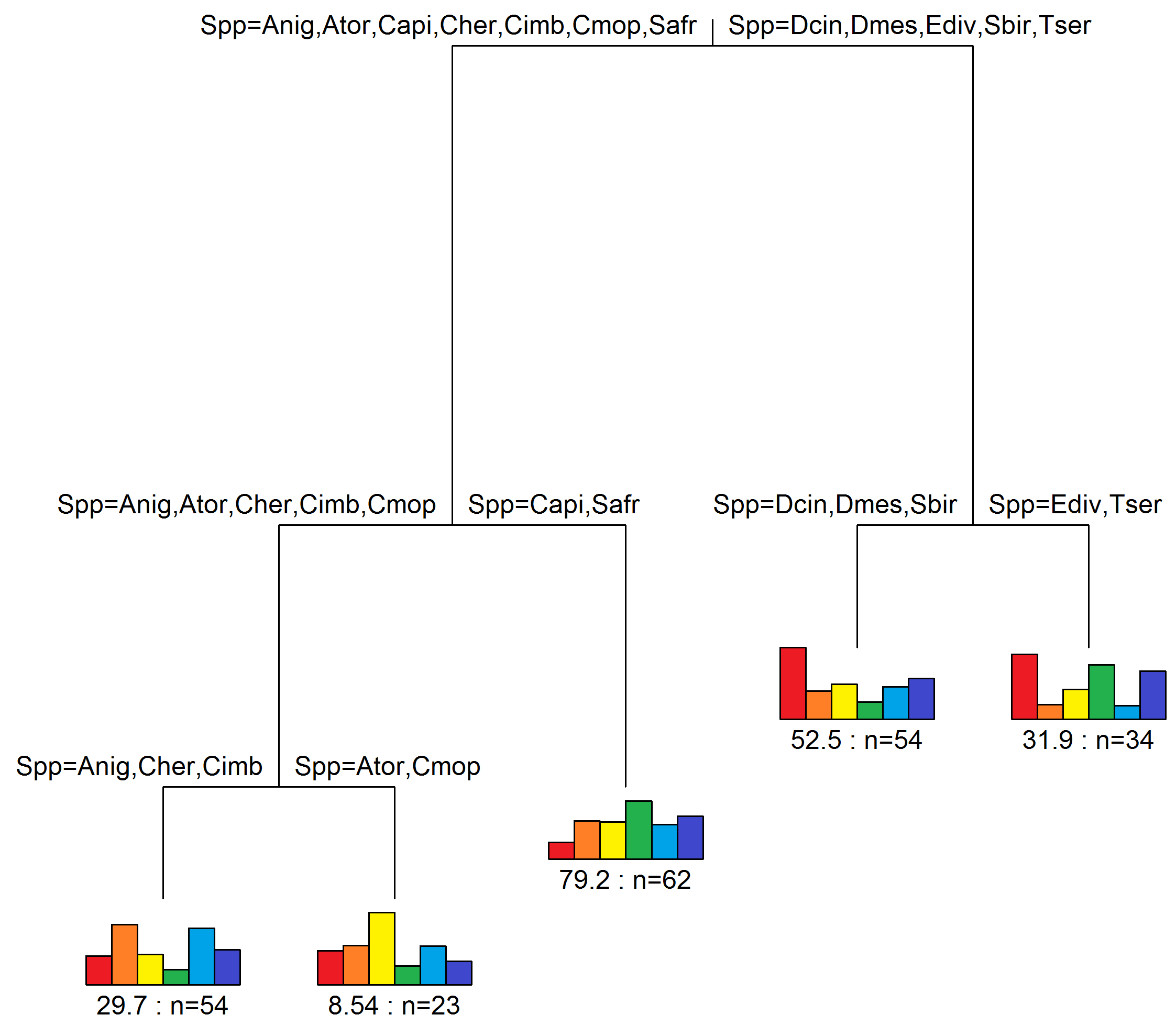

Supplement: S3 Fig — Five groups of species accounted for 54% of the total variance across six chemical traits (lignin, hemi-cellulose (Hemicell), Zn, Mn, B, Mg). Although it was expected that species would have the strongest multivariate correlation to these six chemicals (because they were selected for this reason during the CART analysis in Fig 2), it is interesting to note that the combination of thorn type and dispersal mechanism have a similarly high correlation (46% variance explained; Fig 5). A total of seven predictor variables (hillslope position, precipitation, parent material, elevation, physical defense, dispersal mechanism, species) were the initial input to the recursive regression tree, but species was the strongest predictor (lowest SSE) for all splits. n = the number of individual plants; the value before n is the sum of squared errors (post-normalization) of chemical concentrations for that group. (TIF) [file pone.0124078.s003.tif]
