# Supplementary material for: Tree Foliar Chemistry in an African Savanna and Its Relation to Life History Strategies and Environmental Filters
Source: PLoS One. 2015 May 20;10(5):e0124078. doi: 10.1371/journal.pone.0124078 (PMC4438986; doi:10.1371/journal.pone.0124078)
Supplement: S1 Table — Results of the intra-specific variation portion of analyses of variance (F-values) for leaf traits of canopy populations at Nwaswitshaka, showing differences for within species and hillslope position interactions. Significant differences (p < 0.05) are indicated by the asterisk. (DOCX) [file pone.0124078.s008.docx]

S1 Table.

|  | **A.nig** |  | **C.api** |  | **S.bir** |  |
| --- | --- | --- | --- | --- | --- | --- |
|  | **Species** | **Species x**  **Position** | **Species** | **Species x**  **Position** | **Species** | **Species x**  **Position** |
| **Light & Growth** | | | | | | |
| Chl a+b | 1.6 | 1.4 | 1.3 | -0.3 | 1.5 | -0.2 |
| P | 3.6* | -0.6 | 5.3* | 1.4 | -6.8* | 2.1 |
| N | 11.0* | -0.9 | 5.3* | -0.4 | -7.6* | 0.2 |
| d13C | -3.2* | 0.5 | 0.8 | 0.9 | -2.5* | 0.2 |
| Sol C | -5.8* | 1.6 | 13.9* | -2.3 | 4.1* | 2.0 |
| Water | -6.6* | -0.6 | 1.1 | 0.3 | 4.4* | 0.2 |
| Car | 2.8* | 1.7 | 1.4 | -0.2 | 1.7 | 0.5 |
| **Defense & Structure** | | | | | | |
| Tannins | -10.4* | 2.1* | -1.0 | -1.7 | 7.7* | 1.5 |
| Phenols | -11.5* | 1.7 | 3.5* | -0.7 | 6.3* | 0.5 |
| Lignin | -4.9* | 0.1 | -19.3* | 1.2 | 11.1* | -1.3 |
| Cellulose | 0.2 | -3.2* | 1.7 | 4.8* | -14.5* | -1.2 |
| Hemicellulose | 22.5* | -1.2 | 6.5* | -0.8 | -19.1* | -0.7 |
| C | -5.8* | 0.2 | 8.4* | -0.5 | -3.1* | -0.5 |
| **Maintenance & Metabolism** | | | | | | |
| Ca | 5.4* | 1.2 | -3.6* | -0.3 | 6.9* | 1.6 |
| K | -1.2 | -0.7 | 1.9 | 2.4* | 4.2* | 0.3 |
| Mg | 4.6* | 1.3 | 0.5 | -2.2 | -2.3* | -1.2 |
| Zn | -2.2* | -0.6 | 7.0* | -2.1 | -14.4* | 2.3* |
| Mn | -6.8* | 1.0 | 12.3* | -5.0* | -12.8* | 2.2* |
| B | 19.1* | -0.8 | -4.3* | -0.4 | -4.4* | 2.6* |
| Fe | 5.1* | -1.7 | -4.5* | 0.2 | 4.7* | -1.3 |
